# Group-Wise Herding Behavior in Financial Markets: An Agent-Based Modeling Approach

**DOI:** 10.1371/journal.pone.0093661

**Published:** 2014-04-08

**Authors:** Minsung Kim, Minki Kim

**Affiliations:** 1 Graduate School of Innovation and Technology Management, Korea Advanced Institute of Science and Technology (KAIST), Daejeon, Republic of Korea; 2 Graduate School of Management, Korea Advanced Institute of Science and Technology (KAIST), Seoul, Republic of Korea; Cinvestav-Merida, Mexico

## Abstract

In this paper, we shed light on the dynamic characteristics of rational group behaviors and the relationship between monetary policy and economic units in the financial market by using an agent-based model (ABM), the Hurst exponent, and the Shannon entropy. First, an agent-based model is used to analyze the characteristics of the group behaviors at different levels of irrationality. Second, the Hurst exponent is applied to analyze the characteristics of the trend-following irrationality group. Third, the Shannon entropy is used to analyze the randomness and unpredictability of group behavior. We show that in a system that focuses on macro-monetary policy, steep fluctuations occur, meaning that the medium-level irrationality group has the highest Hurst exponent and Shannon entropy among all of the groups. However, in a system that focuses on micro-monetary policy, all group behaviors follow a stable trend, and the medium irrationality group thus remains stable, too. Likewise, in a system that focuses on both micro- and macro-monetary policies, all groups tend to be stable. Consequently, we find that group behavior varies across economic units at each irrationality level for micro- and macro-monetary policy in the financial market. Together, these findings offer key insights into monetary policy.

## Introduction

John Maynard Keynes, the most influential economist of the 20^th^ century, claims that desirable behavior by an individual can adversely affect the entire system, which is related to the notions of the “fallacy of composition” and the “paradox of thrift” [1]. In other words, individual elements interact, and these interactions are important to the entire system. Concerning such interactions among individuals, many scholars have attempted to describe group behaviors in a wide variety of subjects [2]–[9]. Of these diverse subjects, this study focuses on irrational behaviors, a major issue of research interest in finance because such behavior can cause bubbles and crashes in the financial market. As psychological factors influence the behavior of economic entities, their effects have been studied [10]–[18] in the financial and economics literature including the domains of behavioral economics, behavioral finance, and quantitative behavioral finance. Furthermore, the relationship between policy formulation and irrational behavior [19]–[22] and the characteristics of the financial market [23]–[31] have also been examined.

Although the volatility of the financial market has recently been high, group behavior has caused financial market fragility [32]–[38] throughout history, including the Great Depression (1929), the oil shock (1973, 1979), the real estate bubbles in Japan (1980s), the LTCM bankruptcy and Asian currency crisis (1997∼1998), the IT bubbles (1990s), the real estate bubbles in the U.S. (2000s), the subprime mortgage loan crisis (2008), the eurozone crisis (2010), and the lowered U.S. credit issue (2011). Based on the high asymmetry and uncertainty existing in the financial market causing this fragility, we are interested in interpreting and understanding the correlation between monetary policy and the behavior of economic units, which is necessary to solve the bubbles and crashes in the financial market [39].

In this study, we define herein “irrationality level” as the extent to which economic units attempt to behave irrationally. The micro-level investigation of the financial market [40] emphasizes the importance of micro-level interactions. Moreover, according to crowd psychology, group formation occurs through commonalities [41], and the behaviors of the resulting economic units are homogeneous and biased in the group even though the economic units are themselves heterogeneous. Commonalities are crucial for group formation and the characteristic behavior of each group. Therefore, group formation and the characteristic behavior of each group are powerful variables for analyzing and solving financial problems. In this regard, group formation can be separated according to the irrationality level of a financial problem prevailing in the financial market. Therefore, the shared reactions of economic units differ in response to various monetary policies.

In this paper, we examine the irrational behavior of different groups (as judged by irrationality level) based on their responses to two types of monetary policies and examine their response characteristics by using the Hurst exponent and Shannon entropy. The two types of monetary policies are distinguished by their efficiency levels, which are related to the stabilization of irrational behavior. Highly efficient monetary policy indicates that the stabilization of irrational behavior is high and vice versa.

Our analysis is based on the agent-based modeling (ABM) method, which focuses on the micro-interactions of each economic unit (agent) because these affect the behavior of the macro-structure and cause the system to emerge. The ABM method can handle a much wider range of nonlinear behaviors than conventional models [42], [43], and thus it is used broadly in economics research [44], [45]. In brief, we aim to describe the importance of quantifying group irrationality level when implementing monetary policy.

## Preliminaries

### Analytical Methods

The Hurst exponent [47], which is a popular analytical tool in the financial field [48], [49], shows three characteristic time series types and it ranges from zero to one. For the first type, 0<H<0.5, which indicates an anti-persistent series and which implies that an up value follows a down value and vice versa. For the second type, H = 0.5, which indicates a random series and implies a Brownian motion. For the third type, H>0.5, which indicates a persistent series and implies that the trends tend to be maintained. Therefore, the strength of a persistent series increases as H approaches one and the strength of an anti-persistent series increases as H approaches zero. The Hurst exponent can thus be calculated by using rescaled range (R/S) analysis as follows:
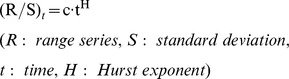



This study employs the Shannon entropy [50], which is an appropriate tool for analyzing the randomness and unpredictability of a system. The Shannon entropy is defined as follows:
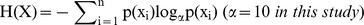



If the value of p(x_i_) is one, the state occurs only once. Therefore, the Shannon entropy is zero. However, if the probability is gradually distributed among states, the Shannon entropy increases. Thus, maximizing the probability corresponds to maximizing the entropy, which also leads to the maximization of randomness and unpredictability.

## Model

### Model Designs

This study designs and constructs a system as follows. Suppose bubbles or crashes happen in the financial market; thus, irrational behavior occurs in each group and for each monetary policy. The variables of interest are the magnitude of the micro-monetary policy (D) and the magnitude of the macro-monetary policy (S), which are the two representative monetary policy types examined herein. D is defined as a concrete, clear, and detailed monetary policy for problems related to economic units. S is defined as a monetary policy whose aim is clear but for which the details for the economic units are blurred and deficient. The criterion for the difference between D and S is the absorbing mechanism of a monetary policy to the crowd for solving problems in a financial market. Therefore, D and S are related to the degree of irrational behavior (N), which is related to the irrationality level. These dynamics cause the emergence of a new system. In addition, the irrationality of the group level is determined by the financial problems related to the group’s irrational actions. Such financial problems are related to commonalities, the formation of the irrationality group, and the cause of the irrational behavior.

As D increases, the system remains stable and irrational behavior decreases. However, when only S is considered instead of D to overcome a system problem, the situation worsens. The irrationality magnitude determines the characteristic group behaviors, which interact with D and S. Further, the total number of agents is 630 and the degree of irrationality is distributed across 13 levels. In addition, the magnitude of monetary policy is distinguished by four levels.

### Modeling Formula

In our model, we design the system by using the exponential function and the work function. The exponential function determines the particular probability of a state. In this study, D effectively reduces group fluctuation, but S must be used to explain the inefficiency of the macro-monetary policy or the probability of irrational behavior occurrences except for D. In addition, the particular probability of a state is proportional to the exponential function as follows.

The exponential function provides us with a specific probability of state t, which is expressed as a selected time flow. Thus, the exponential function of this study can be expressed as exp [θ·(−S/N)]_t_, which is the inefficiency of the macro-monetary policy or the probability of irrational behavior occurring. In addition, θ is constant.

The relationship between S and N determines the particular probability of a state. In particular, when S is greater than N, i.e., S>>N, the inefficiency of the macro-monetary policy approaches zero, whereas when N>>S, the inefficiency of the macro-monetary policy approaches one. Furthermore, according to the photoelectric effect, the work function represents the minimum energy needed to produce an electric current in the metal. The formula of the kinetic energy in the photoelectric effect [46] is expressed as follows:
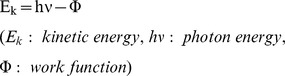



From this formula, the positive difference hν – Φ provides us with the kinetic energy of the electron. Thus, characteristic behaviors can arise when the difference between the action and the resistance is positive. Therefore, the formula used in this study is expressed as follows:



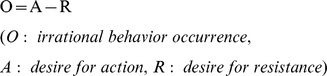



O is considered to be the speculating and dumping actions that produce bubbles and crashes. These phenomena originate from the positive difference between A and R. Moreover, the magnitude of irrationality affects A, and exp [θ·(−S/N)]_t_ is related to R. The characteristic of O is based on the characteristics of monetary policy. Hence, the exponential function and work function concepts used in this work are crucial to understanding the randomness and unpredictability of each group in an economic system.

## Results

**Figure 1:**
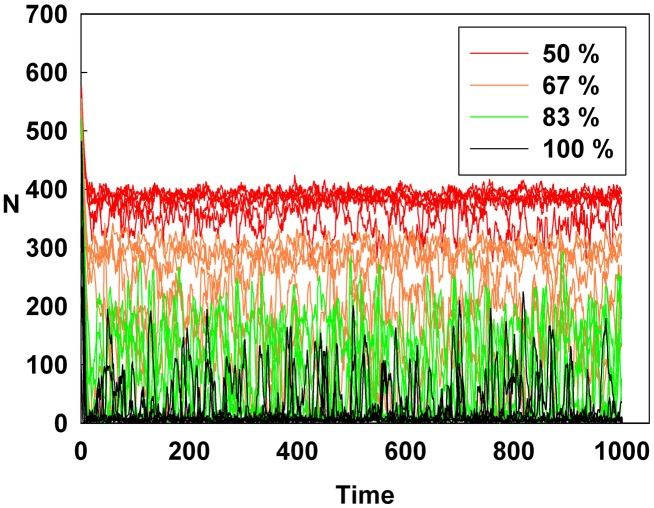
Line colors show the percentage of the maximum D at a constant initial S for the six higher irrationality level groups.

**Figure 2:**
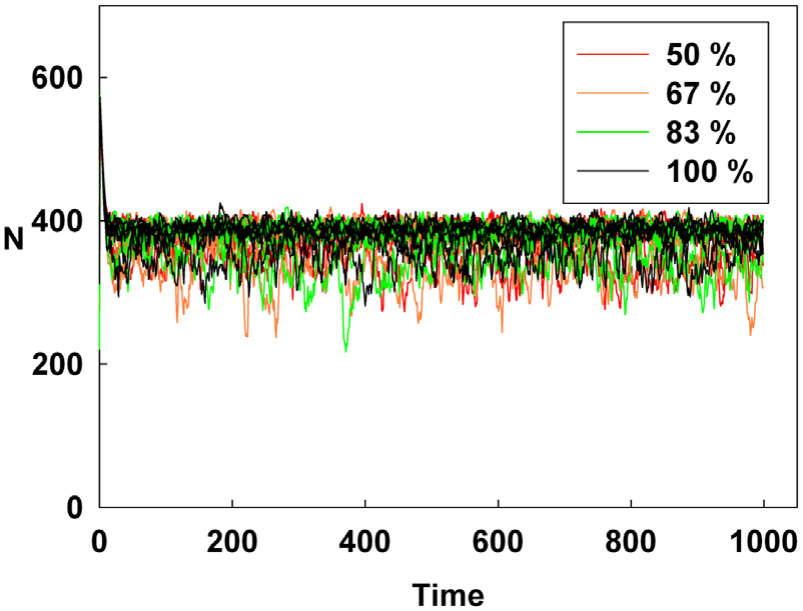
Line colors show the percentage of the maximum S at a constant initial D for the six higher irrationality level groups.

**Figure 3:**
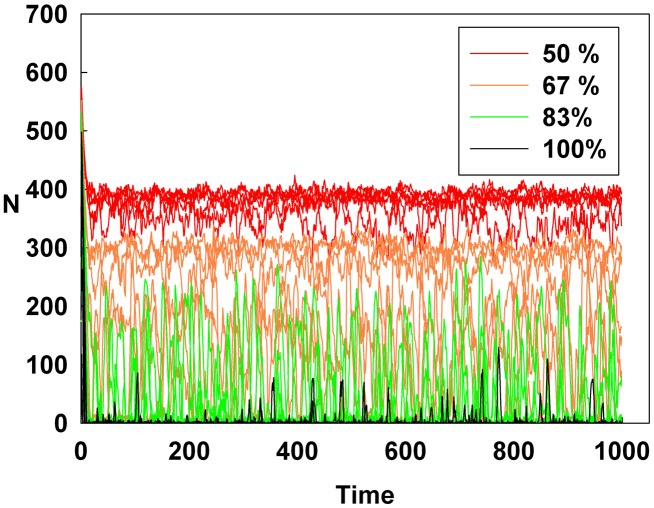
Line colors show the percentage of the maximum D and S for the six higher irrationality level groups.

Figures 1, 2, and 3 show the degree of irrational behavior in the six groups (i.e., those that have irrationality levels above a medium level) with a change in the corresponding monetary policy. In Figure 1, the degree of irrational behavior decreases as D increases for a constant initial S. At the maximum D, the irrational behaviors of the groups are minimized. In Figure 2, although S increases for a constant initial D, the degree of irrational behavior in the groups is unsteady. In this case, the increasing S is meaningless for stabilizing the irrational behavior of economic units. The increasing S does not alter the degree of irrational behavior. In Figure 3, the degree of irrational behavior is effectively decreased by simultaneously increasing both D and S.

**Figure 4:**
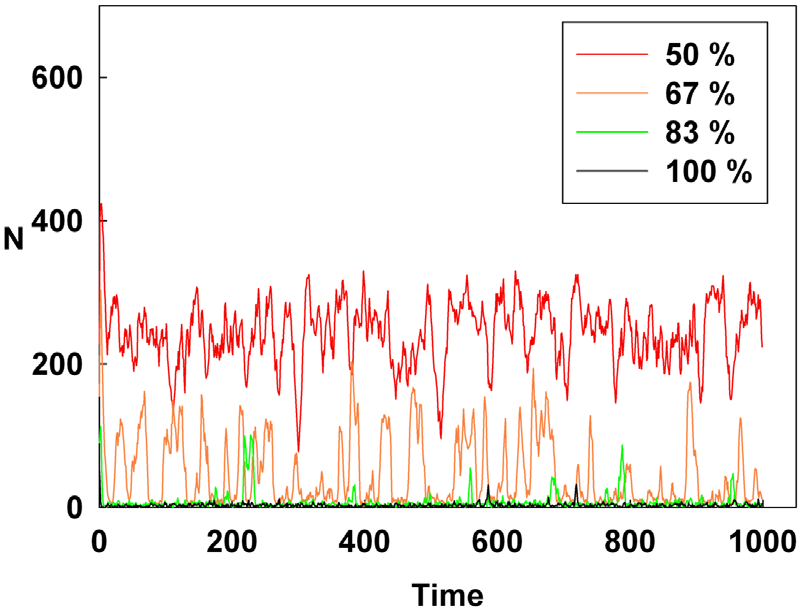
Line colors show the percentage of the maximum D at a constant initial S for the medium irrationality level group.

**Figure 5:**
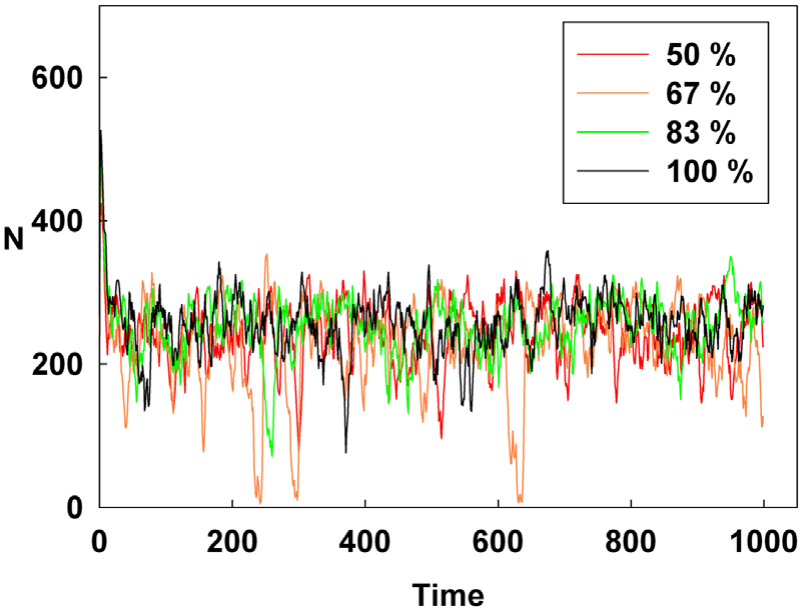
Line colors show the percentage of the maximum S at a constant initial D for the medium irrationality level group.

**Figure 6:**
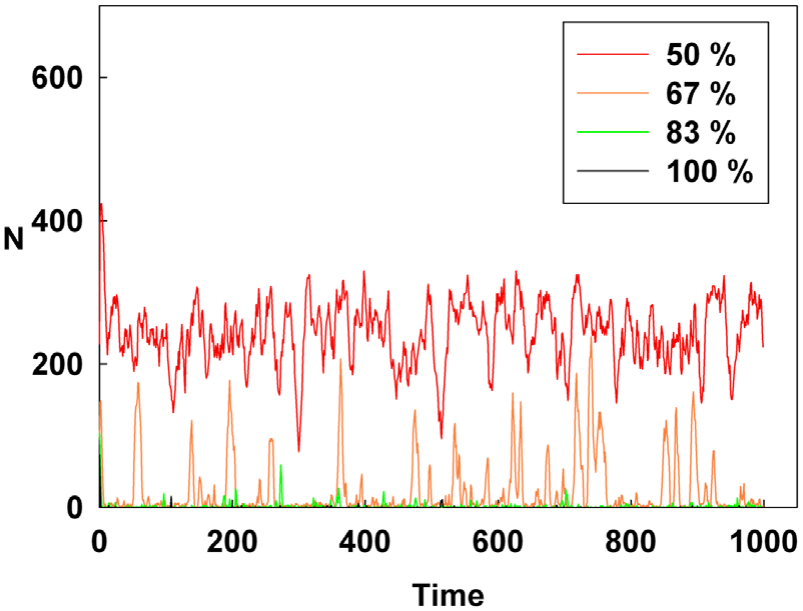
Line colors show the percentage of the maximum D and S for the medium irrationality level group.

Figures 4, 5, and 6 show the degree of irrational behavior for the medium irrationality group only with a change in the corresponding monetary policy. Figure 4 shows that as D increases for a constant initial S, the degree of irrational behavior is smaller than that for the higher irrationality level. In Figure 5, the irrational behavior occurrences are maintained around a specific level by increasing S at a constant initial D. In Figures 2 and 5, the degree of irrational behavior is not minimized by simply changing S from the minimum-level monetary policy to the maximum-level monetary policy. Thus, only changing S does not affect the irrationality groups. In Figure 6, the degree of irrational behavior decreases by simultaneously increasing both D and S, which implies that the volatility and vulnerability of the financial market also decrease.

**Figure 7:**
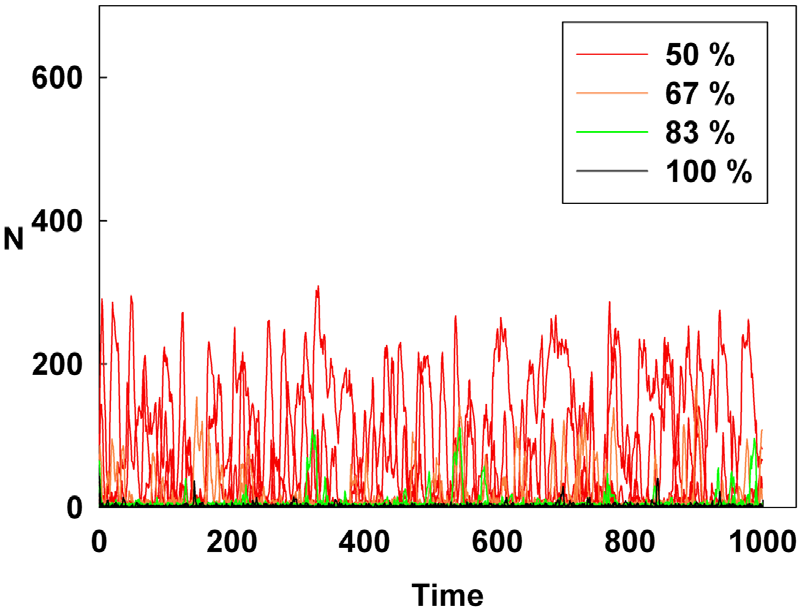
Line colors show the percentage of the maximum D at a constant initial S for the six lower irrationality level groups.

**Figure 8:**
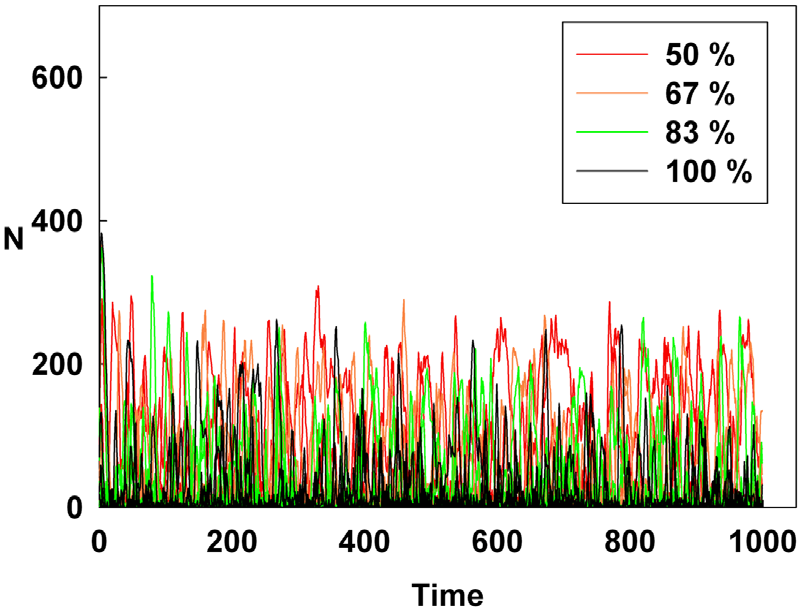
Line colors show the percentage of the maximum S at a constant initial D for the six lower irrationality level groups.

**Figure 9:**
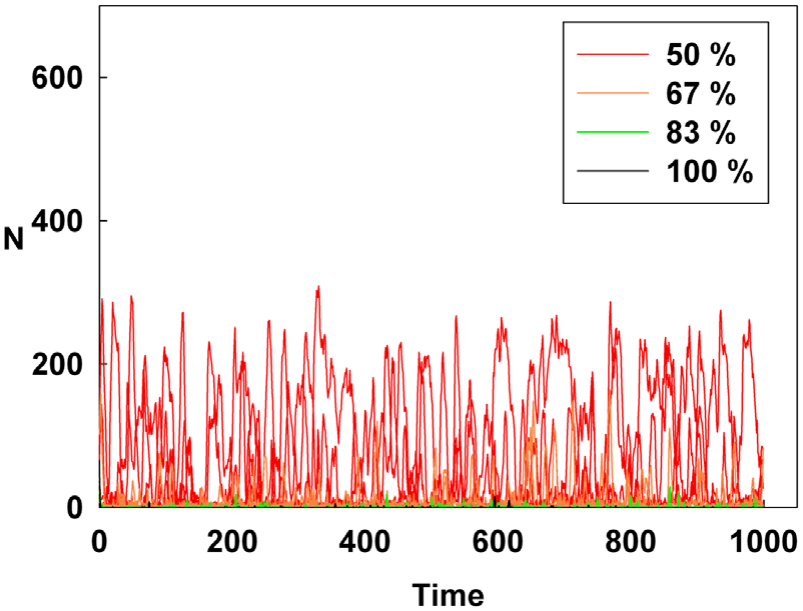
Line colors show the percentage of the maximum D and S for the six lower irrationality level groups.

Figures 7, 8, and 9 show the degree of irrational behavior in six groups (i.e., those that have irrationality levels below a medium level) with a change in the corresponding monetary policy. Figure 7 shows that the degree of irrational behavior in these groups as D increases at a constant initial S. In general, the degree of irrational behavior decreases as the irrationality level decreases. In addition, the degree of irrational behavior decreases with increasing D. Figure 8 shows that the degree of irrational behavior increases with increasing S at a constant initial D. In Figure 9, the degree of irrational behavior in these groups also decreases by simultaneously increasing both D and S.

**Figure 10:**
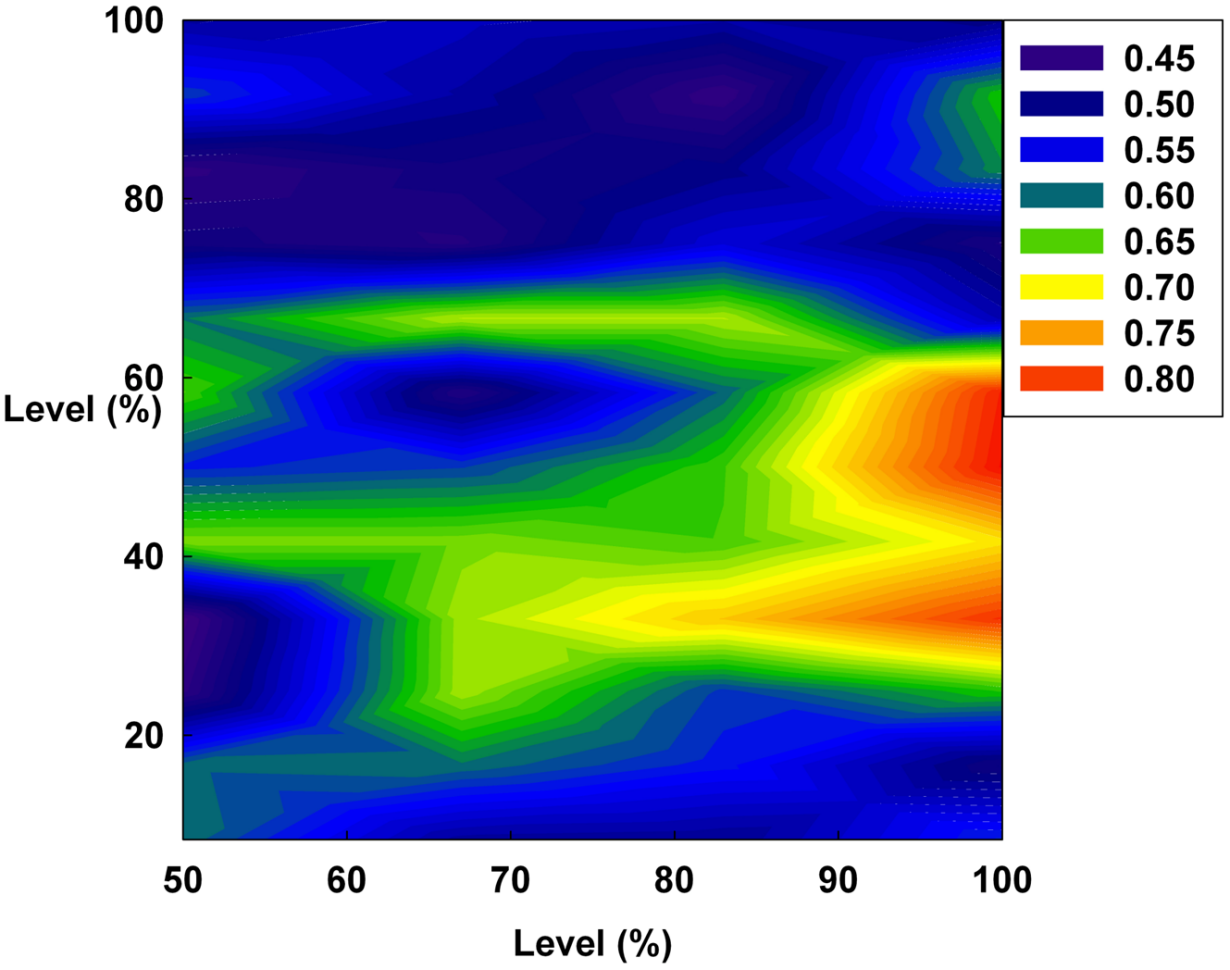
The Hurst exponent value of all groups on a contour. The X-axis indicates D and the Y-axis indicates the irrationality level at a constant initial S.

**Figure 11:**
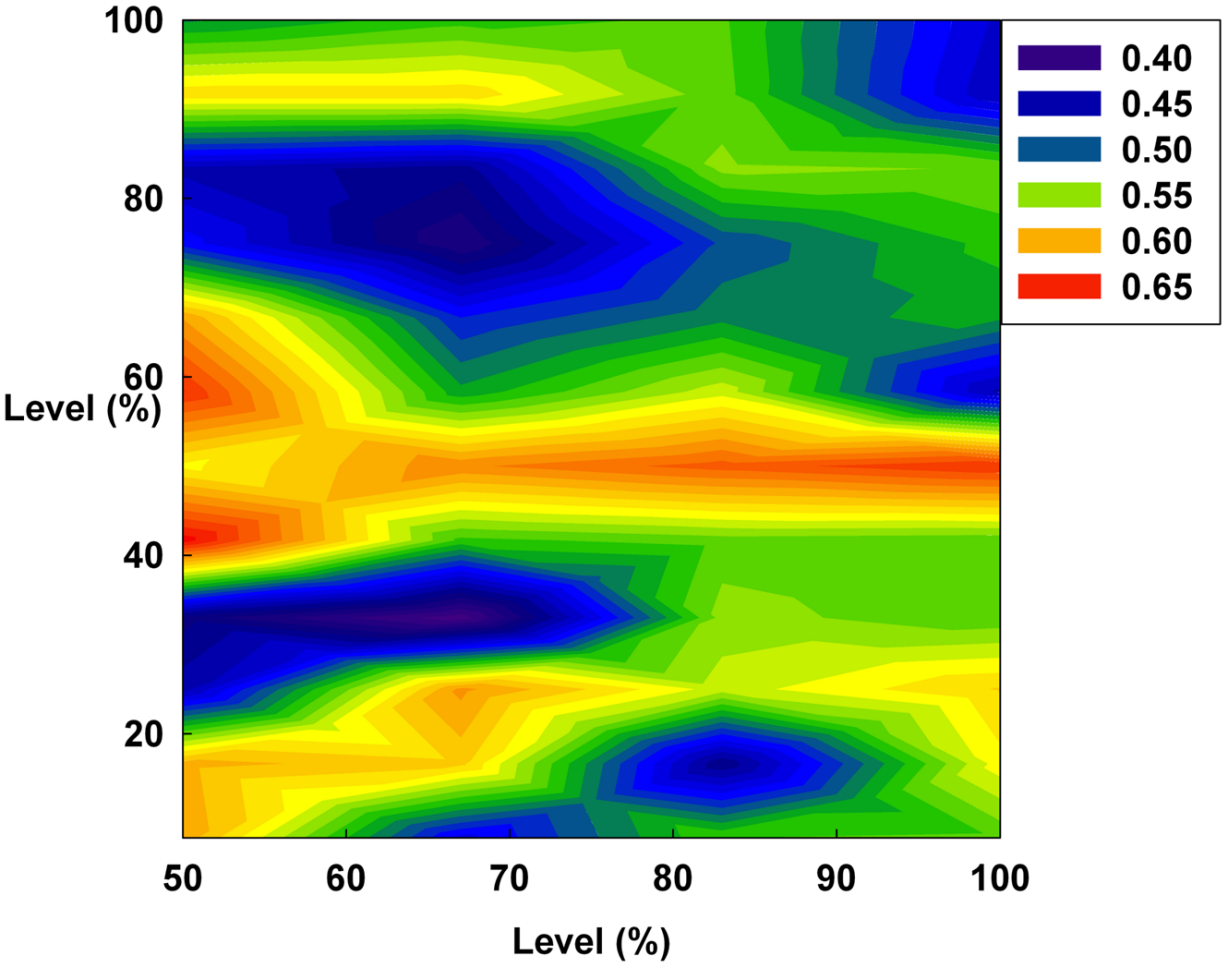
The Hurst exponent value of all groups on a contour. The X-axis indicates S and the Y-axis indicates the irrationality level at a constant initial D.

**Figure 12:**
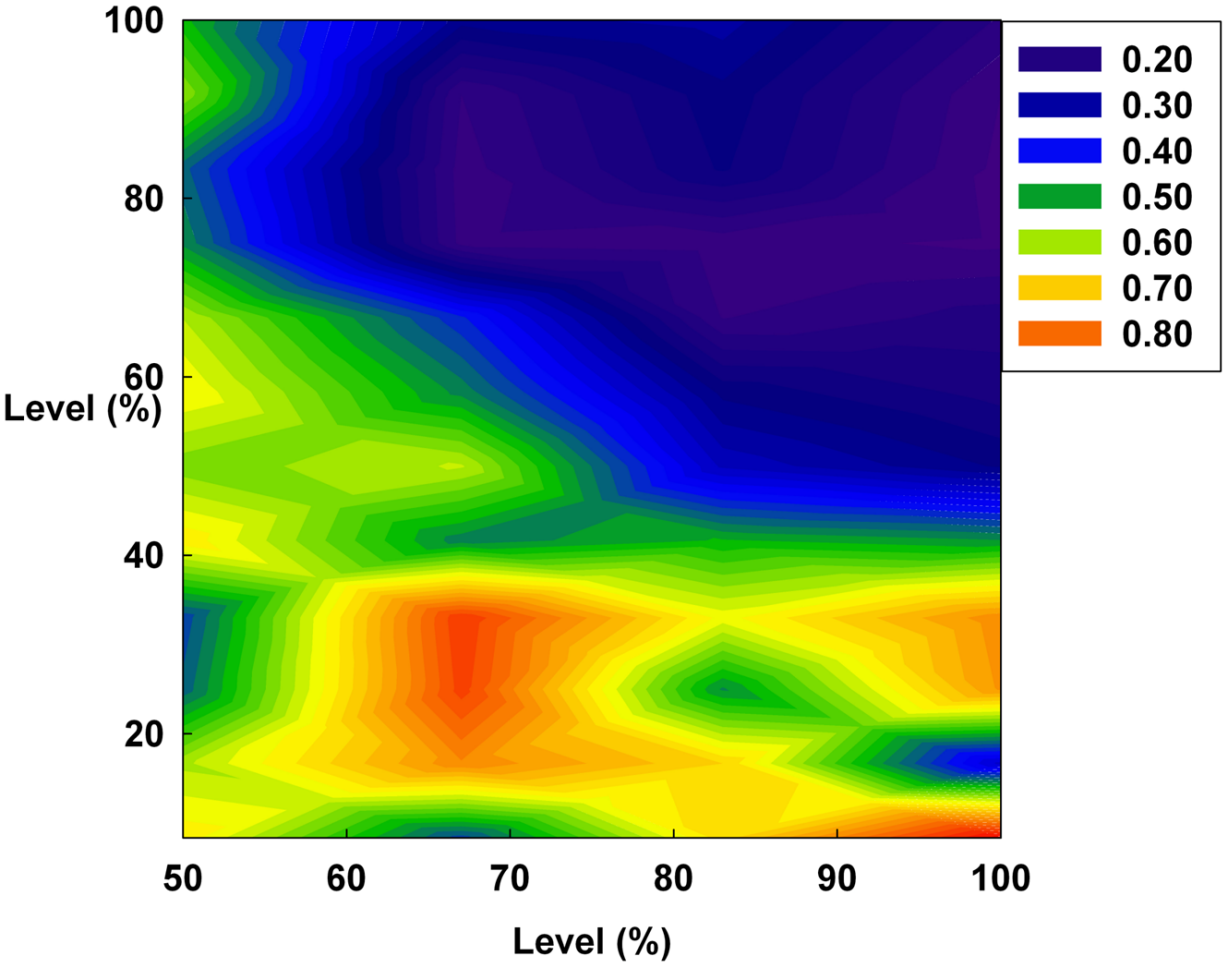
The Hurst exponent value of all groups on a contour. The X-axis indicates the levels of D and S. The Y-axis indicates the irrationality level.

Figures 10, 11, and 12 show the values of the Hurst exponent for all groups on a contour. In Figure 10, the Hurst exponent value of the groups increases by increasing D at a constant S. This result indicates that the strength of the subsequent trends is determined by D. Therefore, by considering the results illustrated in Figures 1, 4, and 7, we find that D makes the system more stable than any other monetary policy. In other words, the groups, which are distributed from the minimum irrationality level to the maximum irrationality level, become strong trend followers as D increases, thereby making the system more stable. Thus, by comparing Figures 1, 4, and 7 with Figure 10, it can be observed that an increased D stabilizes the system by increasing the Hurst exponent value of the groups.

However, in Figure 11, the Hurst exponent value of the medium irrationality group increases as S gradually increases at a constant initial D. Further, at the maximum value of S, the medium irrationality group has the highest Hurst exponent value of all the groups. Figures 2, 5, 8, and 11 show that when only S increases, the medium irrationality group becomes a stronger trend follower and has a smaller Hurst exponent value than the groups in Figure 10, but it still tends to follow the fluctuation trend. Therefore, the fluctuation in irrational behavior does not decrease effectively. Finally, Figure 11 shows that S by itself makes the system chaotic and random. In Figure 12, the Hurst exponent decreases to below 0.5 when both D and S simultaneously increase, which implies that the system changes from a persistent series to an anti-persistent one. Therefore, as shown in Figures 3, 6, 9, and 12, increasing irrational behavior tends to follow decreasing irrational behavior, which stabilizes the system.

In summary, the Hurst exponent value is proportional to the monetary policy magnitude in Figure 10 but inversely proportional to it in Figure 12. However, in Figure 11, the Hurst exponent value is maintained at a specific level regardless of the monetary policy magnitude. In addition, the medium irrationality group is vulnerable and sensitive to all monetary policy characteristics, which implies that this group easily follows a stabilizing or fluctuating trend.

**Figure 13:**
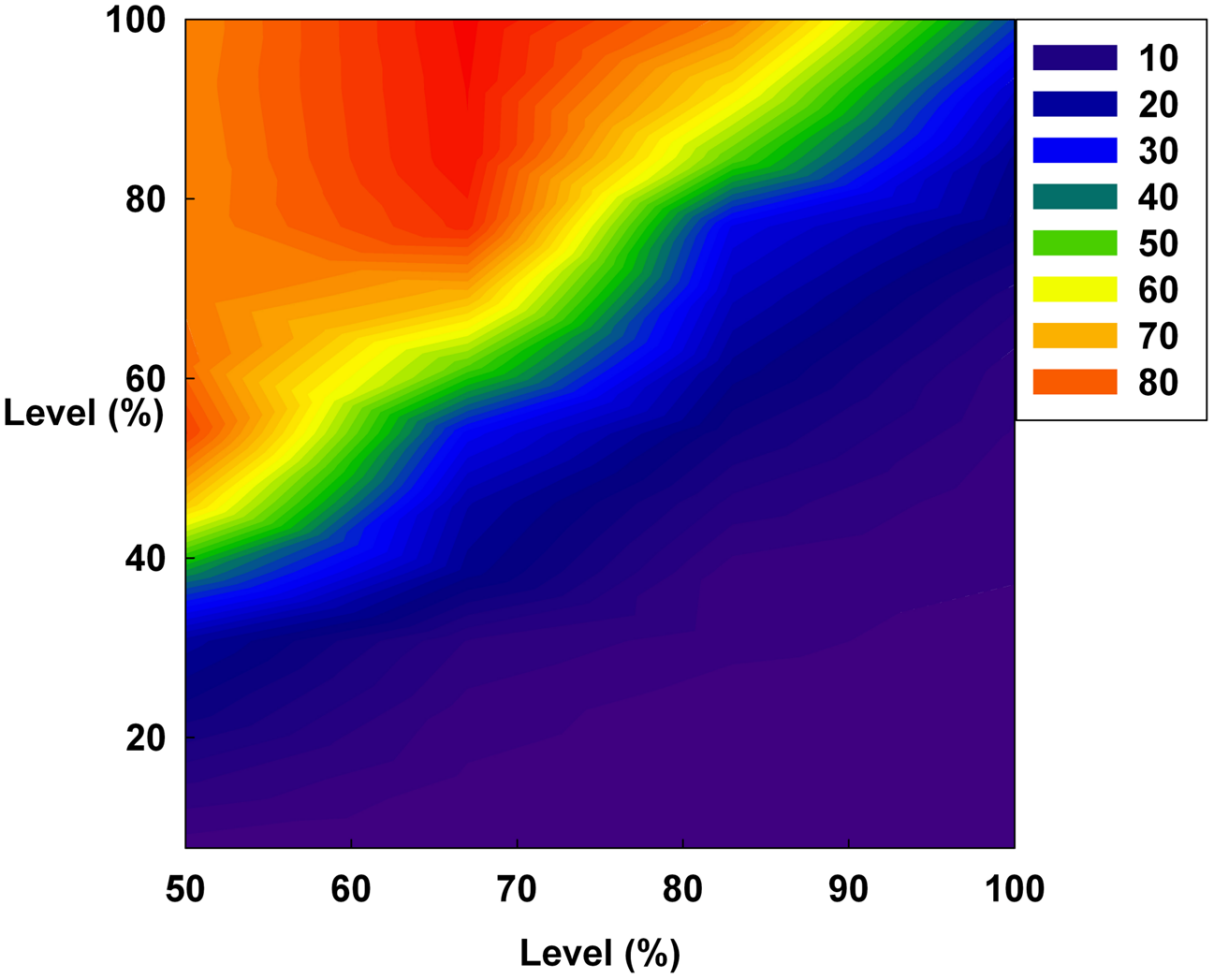
The Shannon entropy level of all groups over all time flows on a contour. The X-axis indicates D and the Y-axis indicates the irrationality level at a constant initial S.

**Figure 14:**
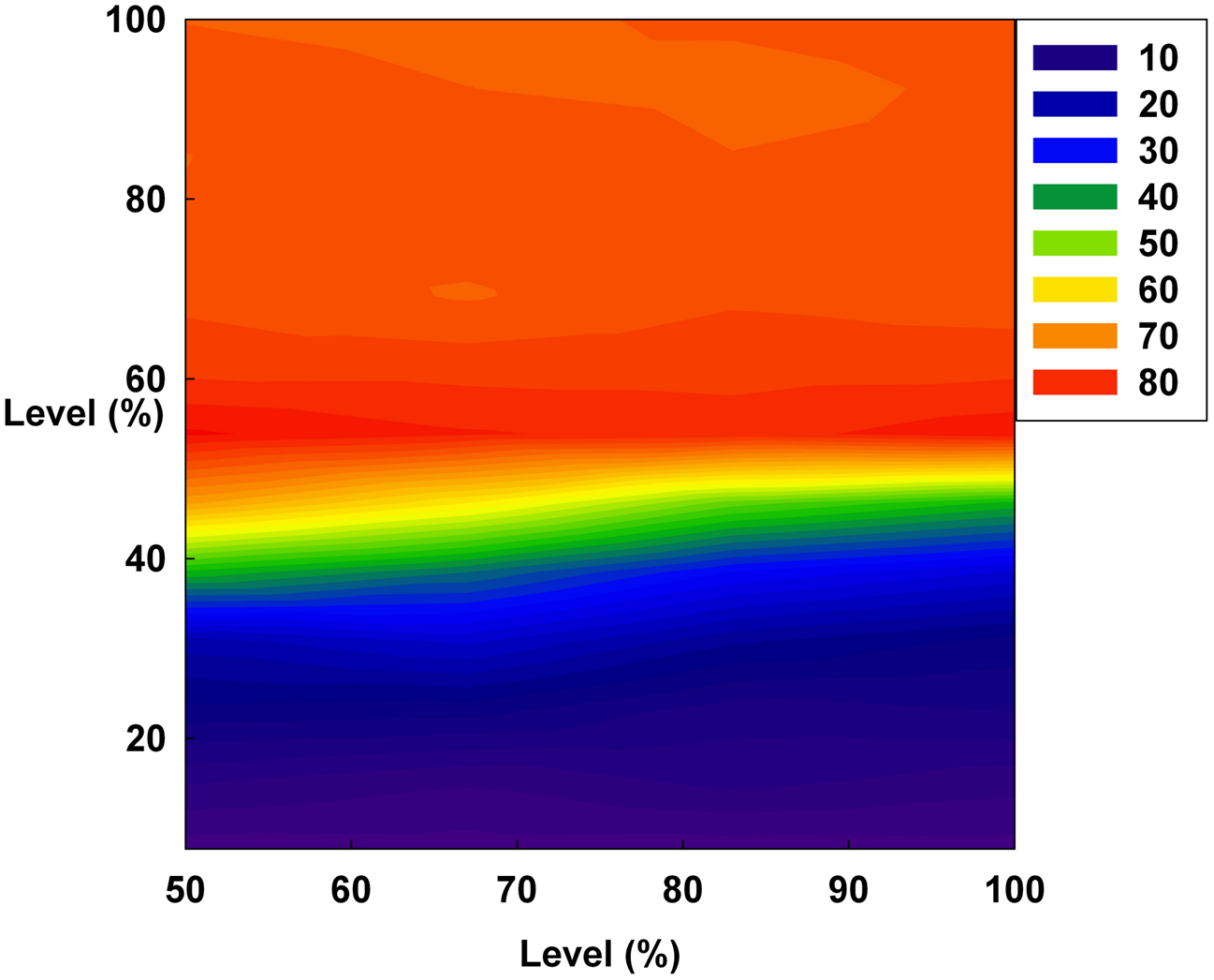
The Shannon entropy level of all groups over all time flows on a contour. The X-axis indicates S and the Y-axis indicates the irrationality level at a constant initial D.

**Figure 15:**
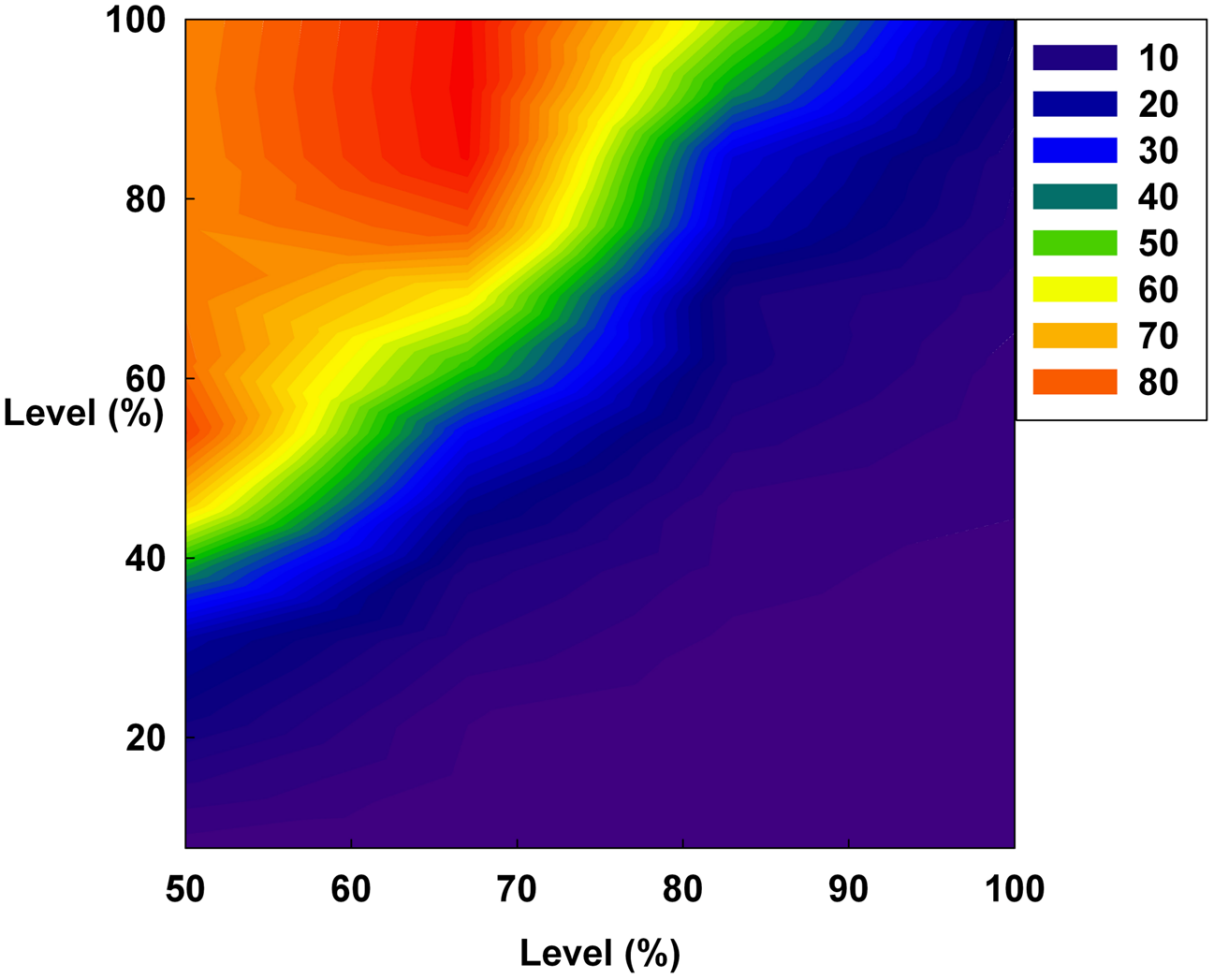
The Shannon entropy level of all groups over all time flows on a contour. The X-axis indicates the levels of D and S. The Y-axis indicates the irrationality level.

Figures 13, 14, and 15 show the average Shannon entropy level of all groups over all time flows on a contour. In Figure 13, the average Shannon entropy level of each group decreases by increasing D at a constant S. Figures 1, 4, 7, and 10 show that the system is stabilized by increasing only D. Further, in Figure 13, an increase in D decreases the randomness of the system and increases the Hurst exponent value.

However, in Figure 14, increasing only S at a constant D does not decrease the average Shannon entropy. Furthermore, in this situation, the medium irrationality group has the highest average Shannon entropy level among all the groups, which is related to the strongly increasing randomness and unpredictability. Thus, in Figures 2, 5, 8, 11, and 14, S is not related to the decreased randomness of the system. Moreover, in these situations, the Hurst exponent values are distributed at approximately 0.5, which implies that the system tends to be random. In summary, when only S increases, the system becomes more unpredictable.

Finally, in Figure 15, the reddish area of the average Shannon entropy is smaller than that in Figure 13, and the Hurst exponent value is notably lowered by simultaneously increasing both D and S. Figures 3, 6, 9, 12, and 15 show that simultaneously increasing both D and S is more effective at stabilizing the system than increasing only D.

## Discussion and Conclusion

Agents in large economic systems are heterogeneous because they display different micro-level motives that result in them being classified into different characteristic groups, which cause fluctuations in the system. This study used the ABM method in order to examine the dynamic behavior of each of these economic units based on different efficiencies of monetary policy. We found that the medium irrationality group, as described herein, has the highest Hurst exponent and Shannon entropy among all the studied irrationality level groups for any monetary policy, which indicates that the behavior of this group shows the highest volatility and vulnerability. From this perspective, if only macro-monetary policy were considered, the medium irrationality group would cause turmoil in the system. However, we also showed that an uncertain and unreliable monetary policy makes the system unstable for all irrationality groups. The presented findings demonstrated the importance of understanding the behavioral characteristics of all economic units in order to solve economic problems. This study also suggested some implications of monetary policy choices.
